# Antimalarial Efficacy of Ethanol Extract of Bridelia micrantha Stem Bark against Plasmodium berghei-Infected Mice

**DOI:** 10.1155/2024/8821019

**Published:** 2024-03-25

**Authors:** Tako Djimefo Alex Kevin, Yamssi Cedric, Noumedem Anangmo Christelle Nadia, Tientcheu Noutong Jemimah Sandra, Mounvera Abdel Azizi, Ngouyamsa Nsapkain Aboubakar Sidiki, Gamago Nkadeu Guy-Armand, Mbohou Nchetnkou Christian, Essangui Same Estelle Géraldine, Tankoua-Tchounda Roméo, Vincent Khan Payne, Lehman Léopold Gustave

**Affiliations:** ^1^Department of Animal Organisms, Faculty of Science, University of Douala, P.O. Box 24157, Douala, Cameroon; ^2^Laboratory of Tropical and Emerging Infectious Diseases, Dschang, Cameroon; ^3^Department of Biomedical Sciences, Faculty of Health Sciences, University of Bamenda, P.O. Box 39, Bambili, Bamenda, Cameroon; ^4^Department of Microbiology, Hematology and Immunology, Faculty of Medicine and Pharmaceutical Sciences, University of Dschang, P.O. Box 96, Dschang, Cameroon; ^5^Department of Animal Biology, Faculty of Science, University of Dschang, P.O. Box 067, Dschang, Cameroon; ^6^Department of Biological Sciences, Faculty of Medicine and Pharmaceutical Sciences, University of Douala, P.O. Box 02701, Douala, Cameroon

## Abstract

**Background:**

The spread of drug resistance is a significant issue, particularly in endemic countries with limited resources. The aim of this study was to evaluate antimalarial and antioxidant activity of *B. micrantha* in order to justify its use in traditional medicine.

**Methods:**

Evaluation of the *in vivo* antimalarial activity of *B. micrantha* was carried out according to the model of the suppressive and curative test of Peters' over 4 days in infected *Swiss albino* mice. Antioxidant parameters and stress were measured after intraperitoneal administration of 1 × 10^7^ infected red blood cells.

**Results:**

At doses of 150 mg/kg, 300 mg/kg, and 600 mg/kg, administration of B. micrantha substantially produced suppression of *P. berghei* infection by 67.75%, 73.46%, and 78.99%, respectively, while 84.64% of the untreated group (1% DMSO) had suppression from chloroquine. The curative test significantly decreased the levels of parasitaemia and death in the treated groups. Furthermore, after *B. micrantha* extract was given to infected mice, a noteworthy increase in total protein, aspartate aminotransferase (AST), and alanine aminotransferase (ALT) was observed. On the other hand, hepatic catalase (CAT) and superoxide dismutase (SOD) productions were considerably greater than that of the healthy control. Mice had considerably lower levels of nonenzymatic antioxidant markers such as glutathione, NO, and MDA showing that the liver was protected.

**Conclusion:**

The infected groups responded favorably to the ethanol extract of *B. micrantha*. This result justifies investigation for its use in Cameroon.

## 1. Introduction

Haemoparasite illnesses continue to be a serious hazard to human health. Malaria remains one of the main health problems in the world, with approximately 627,000 deaths per year [1]. *Plasmodium falciparum*, one of the six kinds of parasites that cause malaria in humans, is responsible for the majority of these deaths. Malaria has a severe impact on many people's development and general well-being, making it a burdensome disease. The principal causes of the disease's global spread are the failure of vector control initiatives and the emergence of treatment resistance to chloroquine and other approved antimalarial medications [2]. Scientific research on medicinal plants, particularly those traditionally used to cure malaria, has been encouraged as a result of this trend [3]. The host provides nutrients that Plasmodium must absorb and transform into other molecules or energy. It needs amino acids for the digestion of the host's hemoglobin or for the synthesis of their proteins from the host.

Nadia et al. [4] have reported a substantial correlation between oxidative stress and malaria due to the strong indication that *P. falciparum* can cause reactive oxygen species (ROS) to be converted into free radicals. The development of systemic problems in hosts induced by malaria infection has been largely attributed to the formation of reactive oxygen species (ROS) that generate oxidative stress [5, 6]. Many different substances with artificial antioxidant capabilities have been employed to lower ROS production and shield proteins, lipids, and DNA from oxidative stress-related damage. According to Nosten and White [7], a medication with antimalarial and antioxidant quality increases survival rates, slows the emergence of resistance, and may even stop the spread of parasites that are resistant to treatment. Therefore, a single medication having antioxidant and antiplasmodial properties that can suppress malaria at the same time may be preferable to a therapy with just one action (antimalarial).

The bark of the Cameroonian medicinal plant *Bridelia micrantha* is used to treat malaria by the population in the Noun Division of the Western Region of Cameroon. Antiplasmodial antioxidant activity and *in vitro* cytotoxicity on red blood cells (RBC) and macrophage cells have been demonstrated by [8]. Because of the possibility of the extract being physiologically active *in vitro* and inert *in vivo* as a result of biotransformation and bioavailability processes, it is crucial to conduct an *in vivo* investigation on *B. micrantha*. Therefore, in order to scientifically support the use of *B. micrantha* in traditional medicine, it is crucial to assess its antimalarial and antioxidant activity in *Swiss albino* mice infected with *Plasmodium berghei* ANKA 65.

## 2. Material and Methods

### 2.1. Collection and Identification

The stem bark of *Bridelia micrantha* was collected in Foumban, in the West Region of Cameroon, in 2021 (latitude: 5.71667, longitude: 10.9167). Referencing number 64129/HNC to the voucher specimen, the leaves, seeds, and flowers were sent to the National Herbarium of Cameroon for identification.

### 2.2. Preparation of Plant Extracts

One hundred grams of powder was measured using an electric balance (SF-400) and introduced into 1 L of 95% ethanol. The concentration is 10% g/ml. The mixture was homogenised for 72 h (every 4 hours, the extract was stirred) and filtered using Whatman paper no. 3. The resulting filtrate was dried in an oven at 45°C until the dry extract was obtained.

### 2.3. Plasmodium Strain Used

The strain Plasmodium *berghei* ANKA 65 obtained from the ATCC in the United States of America was used. Our strain was maintained in the laboratory on mice that were kept in cages with varying degrees of ventilation and were given food and water on a daily basis.

### 2.4. Antimalarial Activity

#### 2.4.1. Suppressive Tests

To assess the suppressive effect of *Bridelia micrantha* extract, the Peters 4-day suppressive test [9] was used. Six groups of six albino mice, each weighing between 20 and 30 grams, were assembled from the 36 altogether. Intraperitoneally, on day D0, 200 *μ*l of 1 × 10^7^ parasitized red blood cells was administered to five groups of mice. We used a concentration of 75 g/l for an average weight of 25 g. The first three groups were given dosages of 150, 300, and 600 mg/kg of body weight of the ethanolic extract of *Bridelia micrantha* (D0-D3) three hours after infestation. Each of the final three groups received distilled water (neutral control), 5 mg/kg of chloroquine (positive control), and 1% DMSO (negative control). On a blood smear, each mouse's parasitaemia was assessed starting on the fifth day (D4). Blood was drawn on day 10 in order to measure the hematological parameters. Blood was drawn on day 10 in order to measure the hematological parameters. The following formula was used to determine the mean parasitaemia, percentage of inhibition, and mean survival rate (MSR):

Parasitaemia (*P*):

(1) $$\begin{array}{c} P=\frac{\text{number of parasitized red blood cells}}{\text{total number of red blood cells counted}}\times 100. \end{array}$$

Percentage of inhibition:

(2) $$\begin{array}{c} \%\text{inhibition}=\frac{\text{parasitaemia of the negative control}-\text{parasitaemia of the control test}}{\text{parasitaemia of the negative control}}\times 100,\\ \text{Mean survival rate}=\frac{\text{number of day survived}}{\text{total number of days of mice}}\times 100. \end{array}$$

#### 2.4.2. Curative Tests

**Figure 1:**
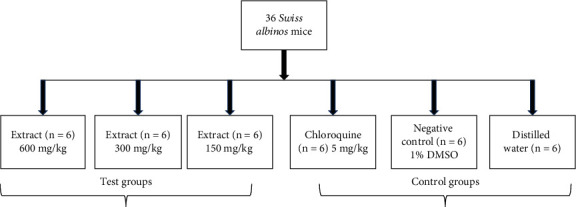
Experimental design.

As shown in Figure 1, thirty-six (36) albino mice (male and female) with an average weight of 25 ± 0.5 g were divided into six groups. Intraperitoneally, on day (D1), 1 × 10^7^ parasitized red blood cells in 200 *μ*l were administered to five (5) groups of these animals. On days 4, 5, 6, and 7, seventy-two hours (72 h) after infection, the experimental animals received treatment. Day 4 marked the determination of parasitaemia using the May-Grünwald-Giemsa staining, as well as the mean survival rate of the treatment groups (D1–D30). The mice that were killed during the stress and hematological tests were excluded from the survival rate calculations. Since chloroquine is still used to treat severe malaria, we used it as a positive control.

*(1) Analysis of Biochemical Parameters*. On day ten of the experiment, the mice were put to death. Next, blood was collected. The serum was used for the dose of AST (aspartate amino transferase) and ALAT (alanine amino transferase), using the Dutch Diagnostic Kit. The blood was centrifuged at 3000 rpm for 10 minutes. After crushing the liver, it was centrifuged for 30 minutes at 3000 rpm in order to assess cell susceptibility to various parameters, including MDA (malondialdehyde), GSH (glutathione), NO (nitric oxide), protein, SOD (superoxide dismutase), and catalase [10–14]. A spectrophotometer (BIOBASE BK-D560) was used to analyze these values. Using a Urit 5160 hematological analyzer, hematological parameters were also evaluated.

### 2.5. Qualitative and Quantitative Screening

Azizi et al.'s approach [15] was used to assess the total phenolic and flavonoid content, while the conventional methods [16] were followed to establish the classes of compounds present in the extracts.

### 2.6. Ethical Approval

The authors hereby certify that they have complied with all applicable national laws as well as the “Principles for the care of Laboratory Animals” (NIH Publication No. 85-23, revised 1985), which include housing, bedding, and feeding considerations for lab animals [17]. The Department of Animal Organisms, Faculty of Science, University of Douala, evaluated and approved each experiment.

### 2.7. Statistical Analysis

ANOVA test followed by Tukey's multiple comparison test was used to compare the treated groups with the control group; values were considered significant at *p* < 0.05. The data was analyzed using GraphPad 8.4 Prism 2, and the result was expressed in the form of graphs and tables. Each sample was run in triplicate.

## 3. Results

### 3.1. Overall Survival Rate, Parasitaemia Level, and Antimalarial Suppressive Effects

Table 1 displays the average survival rate, the level of parasitaemia, and the suppressive impact. This table's analysis demonstrates that, in a dose-dependent way, the extracts of B. micrantha and chloroquine greatly decreased the parasitaemia as compared to the negative control (1% DMSO). The suppression rate for chloroquine was the highest, at 84.64%, compared to 78.99% for the 600 mg/kg dose. The mean survival rate did not differ significantly between the groups treated with extract and those treated with chloroquine.

### 3.2. Curative Test

**Figure 2:**
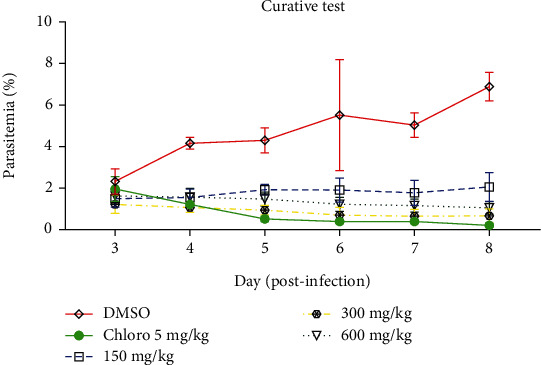
*Bridelia* micrantha ethanol extract's curative properties.

The healing properties of *B. micrantha*'s ethanol extract are displayed in Figure 2. Comparing the ethanol extract of *B. micrantha* to the negative control group (1% DMSO) on days 4–8, this figure demonstrates a substantial dose-dependent curative antimalarial effect (*p* < 0.05) at dosages of 300 mg/kg and 600 mg/kg. Furthermore, on day 7, the lowest dose (150 mg/kg) had an impact, and on day 8, the infection reappeared.

#### 3.2.1. Mean Survival Rate following *B. micrantha* Treatment during the Curative Test

Table 2 displays the average survival rate of *P. berghei-*infected mice. The group treated with chloroquine (5 mg/kg) and the extract doses of 300 mg/kg and 600 mg/kg had an average survival rate of 23.47 and 22.34, respectively, whereas the untreated group (negative control) had the lowest average survival rate among the treatment groups.

#### 3.2.2. Hematological Parameters

The effect of ethanol extract on hematological parameters is displayed in Table 3. This table suggests that although not significant (*p* > 0.05), decreases in WBC count were seen, with the nontreated group experiencing the biggest decrease. The RBC value was statistically significant (*p* < 0.05) for the nontreated group.

#### 3.2.3. Biochemical Characteristics

Table 4 displays the effect of B. micrantha on biochemical variables. After 7 days of infection, *P. berghei* infection in Swiss mice led to significant and noteworthy increases in blood AST, ALT, and liver protein levels compared to the healthy control group. Total protein levels in the infected groups appeared to be significantly (*p* < 0.05) higher than in the normal control, with the exception of the 300 mg/kg and 600 mg/kg doses, which did not differ significantly from the negative control (DMSO).

#### 3.2.4. Enzymatic Antioxidant Parameter

**Figure 3:**
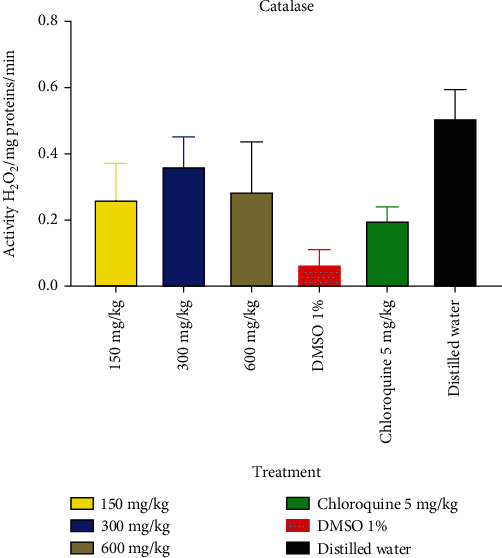
Effect of *B. micrantha* ethanol extract on catalase.

The effect of *B. micrantha* ethanol extract on catalase is depicted in Figure 3. This figure's analysis reveals a substantial difference between the negative control group (which got 1% DMSO) and the normal control group. In the groups that received varying treatment dosages (5 mg/kg, 150 mg/kg, 300 mg/kg, and 600 mg/kg), there was an increase in the production of catalase.

**Figure 4:**
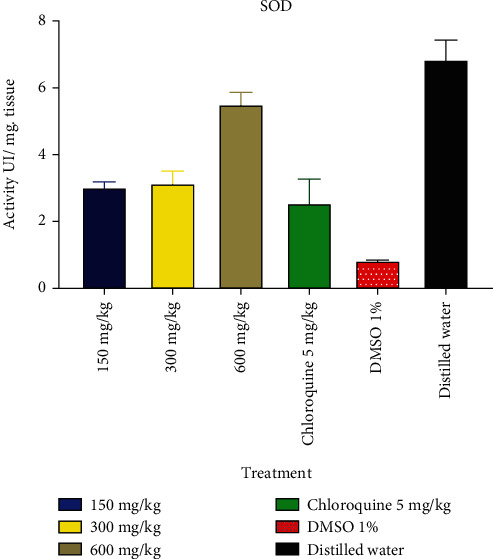
Effect of *B. micrantha* ethanolic extract on superoxide dismutase.

The impact of *B. micrantha* ethanol extract on SOD is depicted in Figure 4. The groups that received therapy had greater SOD rates than the groups that received 1% DMSO, according to the study of this figure.

#### 3.2.5. Enzymatic Activity

Table 5 displays the effects of B. micrantha on NO, MDA, and glutathione. Table 4 demonstrates that the tissues of the negative control (1% DMSO) had higher levels of MDA and glutathione than both the normal control and the various test groups that received the doses (150 mg/kg, 300 mg/kg, and 600 mg/kg). When B. micrantha ethanol extract was given to the animals at varying dosages, the animals' hepatic NO level decreased in comparison to the negative control (1% DMSO).

### 3.3. Total Content of Flavonoids and Phenols

**Figure 5:**
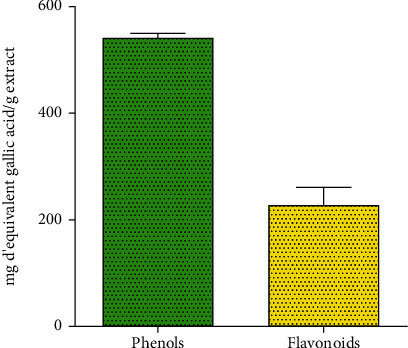
Total content of flavonoids and phenols.

The total phenolic and flavonoid contents of B. micrantha are depicted in Figure 5, from which the phenolic and flavonoid concentrations in the ethanol extract were 541.4 ± 19.81 and 227.8 ± 81.15 mg/g, respectively.

## 4. Discussion

This study has shown that *B. micrantha* ethanol extract significantly decreases parasitaemia in mice infected with *P. berghei*. The suppressing activity of this extract was observed to be dose-dependent, increasing with increasing extract concentration. A substance is declared active if it reduces parasitaemia by at least 30% according to [18]. Previously, *in vitro* activity of *B. micrantha* plant bark extract against *P. falciparum* has been reported [8], suggesting that the plant may possess antimalarial compounds. The fact that the extracts increased the mean survival time suggests that they repressed *P. berghei* and lessened the parasite's overall pathologic impact on the mice. This might be caused by *P. berghei* parasites reappearing after an apparent cure. Our findings about mean survival time are consistent with research on extracts of *Boscia angustifolia*, *Momordica foetida*, and *Nigella sativa* by Muthaura et al. [19], Waako et al. [20], and AbdulElah and Zainal-Abidin [21].

In the curative test, the antimalarial effect of the ethanol extract of the stern bark of *B. micrantha* on the established malarial infection showed a significant chemosuppression (*p* < 0.05) of the parasitaemia, with a maximum dose of 300 mg/kg body weight. Chloroquine at 5 mg/kg body weight did not show 100% eradication of parasitaemia but rather inhibited 75%. An upsurge is observed from day 7 onward. This can be explained by the fact that the plasmodia would have developed better and better adapted to their environment [21]. The longest mean survival time of mice was strongly associated with maximal inhibition of parasitaemia, which was consistent with other *in vivo* antimalarial assays [21]. The mortalities recorded in the groups receiving the treatments are not totally linked to hyperparasitaemia but to an immunopathology that promotes the accumulation of immune cells in the brain and the dysfunction of the organ and leads to symptoms such as paralysis and head deviation [22]; this is in line with the results obtained by Mulisa et al. [18], who recorded 100% death in the untreated group on the 10^th^ day. Some traditional plants showed some antiplasmodial activity in the curative test with prophylactic activity against the *P. berghei* parasite with minimal effect.

Red blood cell and white blood cell hematological characteristics are typical biomarkers of malaria infection and combined indications against malaria infection. The findings revealed significant increases in the untreated group compared to the healthy control group as well as decreases in the number of red blood cells, Hb, MCV, MCH, and MCHC. This shows that after contracting *P. berghei* [23, 24], infected mice experienced severe haemolysis and hypochromic microcytic anaemia. According to [24], this may be caused by the fast lysis of parasitized and healthy red blood cells, the inhibition of bone marrow and erythropoiesis, and dyserythropoiesis. In addition, it has been noted that in mice infected with *Plasmodium berghei*, an increase in red blood cell fragility causes haemolysis and then anaemia [25]. Proinflammatory cytokines like interferon (IFN), tumor necrosis factor (TNF), macrophage migration inhibitory factor, and hemozoin have been linked to the pathogenesis of haemolysis and anaemia during malaria infection, according to a number of studies. Pathogenesis of haemolysis and anaemia is induced by reticulocytosis reduction, the resistance to erythropoiesis, and the inhibition of erythroid precursor proliferation cause by erythropoietin [26, 27]. Furthermore, malaria infection has elevated immunoglobulin-G autoantibodies against uninfected red blood cells, reducing the deformability of red blood cells and enhancing erythrophagocytosis [28]. As part of its anti-inflammatory effects, chloroquine has reportedly been shown to raise erythropoietin levels [29].

WBC participates in leukocytosis and antibody synthesis, which help the immune system fight against foreign antigens. In mice infected with *P. berghei* therapy, white blood cell counts, neutrophil and monocyte counts, and lymphocyte counts significantly increased, whereas lymphocyte counts significantly decreased. These findings imply that infection-induced immune system activation may have evolved as a natural reaction. Neutrophils and monocytes first responded to malaria infection by increasing their phagocytosis activity, while lymphocytes further reduced it [30]. After phagocytic activity has occurred, lymphocytes play a critical function in the immune system by producing antibodies against malaria parasites [31]. Additionally, basophils and eosinophils, which mediate inflammatory and cytotoxic processes related to malarial infection, were significantly elevated in the untreated group. Basophils are key players in proinflammatory responses and are attracted to sites of inflammation [32].

The primary functional organ where ALT and AST activities occur is the liver. According to studies by Jodynis-Liebert et al. [33], Akanbi [34], and Sidiki et al. [35], mice infected with *P. berghei* and left untreated displayed hepatomegaly and an increase in AST and ALT liver enzymes, indicating impairment of some hepatic metabolic functions. These mice also showed hepatocyte leakage as a result of parasite damage. Additionally, we observed decreased protein activity (AST, ALT) in the treated group when compared to the untreated group, which suggests that our extract has hepatoprotective properties. Its possible protective effect against liver damage brought on by *P. berghei* was highlighted by the decrease in enzyme activity brought on by oral administration of *B. micrantha* extract.

According to Tjahjani et al. [36], oxidative stress is a significant clinical and biochemical factor in disease etiology that is becoming increasingly relevant. It happens as a result of the parasites' fast metabolic rate, which causes them to produce a lot of poisonous, redox-active byproducts at a rapid rate of growth and multiplication. This study's observation of an increase in MDA values in infected mice is similar to the findings of Rodrigues and Gamboa from 2009 [37]. Reactive oxygen species (ROS), which are cellular outlaws and can cause havoc in biological systems by harming tissues, changing biochemicals, corroding cell membranes, and even killing, are implied by a rise in MDA levels [38]. This assertion was reinforced by the fact that infected mice had lower levels of SOD, CAT, and GSH activity in their erythrocytes and livers, suggesting that too much ROS inactivates these antioxidant enzymes. This observation is in line with those made by Ibrahim et al. [39], who connected elevated ROS production with decreased SOD and CAT activity in *P. berghei* infection. However, treatment with *B. micrantha* extract boosted the antioxidant enzyme activity. It seems that the flavonoid reduced the amount of lipid peroxidation caused by *P. berghei* and/or raised the amount of substrate (GSH) needed for detoxification by maintaining low ROS levels. Infected mice's antioxidant state was restored, thanks to the *in vivo* antioxidant properties of *B. micrantha* bark extract, which would presumably offer more protection for cell membrane components.

## 5. Conclusion

Mice infected with *P. berghei* responded favorably to the leave extract of *B. micrantha*. The level of certain enzymes, metabolites, or proteins can be affected by malaria, and despite treatment, there has been an alteration of certain metabolic structures as well as a decrease in enzymatic activity after the administration of an ethanolic extract of *B. micrantha* by mouth. However, in order to support the plant's traditional use in Cameroon, *in silico* and toxicological tests are required.

## Figures and Tables

**Table 1:** Mean survival rate, parasitemia level, and suppressive effects.

Treatment	Dosage	Parasitemia	% suppression	Mean survival rate
CQ	5 mg/kg	4.472 ± 2.982^b^	84.64	27.17 ± 1.641^a^

Ethanol extract	150 mg/kg	11.29 ± 4.273^a^	67.75	18.72 ± 3.20
	300 mg/kg	8.975 ± 3.804^a^	73.46	25.35 ± 2.701^a^
	600 mg/kg	7.542 ± 3.173^a^	78.99	26.71 ± 2.142^a^

Negative control	1% DMSO	29.10 ± 13.42^c^	—	8.667 ± 1.801^b^

The data are shown as mean ± SD (*n* = 3); values with the same superscript letter do not differ substantially at *p* ≤ 0.05.

**Table 2:** Mean survival rate of curative test.

Treatment	Dosage	Mean survival rate
CQ	5 mg/kg	25.33 ± 3.127^b^

Ethanol extract	150 mg/kg	16.36 ± 2.214^a^
	300 mg/kg	23.47 ± 2.108^b^
	600 mg/kg	22.34 ± 2.76^b^

Negative control	1% DMSO	7.5 ± 1.384^a^

Values are given as mean ± SD. Values carrying the same superscript letters are not significantly different at *p* < 0.05.

**Table 3:** Effect of the extract during the suppression test on the hematological parameters.

		Red blood cells						White blood cells					
Treatment	Dosage	RBC	Hb	HCT (%)	MCV (fl)	MCH	MCCH	WBC	LYM	NEU	BASO	EOS	MON
Negative control	1% DMSO	2.6 ± 0.9^b^	7.3 ± 4.4^a^	15.6 ± 9.9^a^	47.1 ± 4.6^a^	21.9 ± 1.7^a^	47.1 ± 2.5^a^	4.3 ± 0.7^b^	65.61 ± 6.5^a^	3.6 ± 5.04^a^	0.44 ± 0.7^a^	0.4 ± 0.3^b^	29.94 ± 12.7^a^

CQ	5 mg/kg	6.3 ± 0.4^a^	13.5 ± 1.2^a^	29.7 ± 2.3^b^	47.2 ± 0.2^a^	21.3 ± 0.4^a^	45.3 ± 0.8^a^	20.3 ± 12.9^a^	44.57 ± 4.2^b^	18 ± 6.5^b^	1.21 ± 0.2^a^	1.38 ± 1.2^b^	34.7 ± 9.5^a^

Ethanolic extract	150 mg/kg	4.1 ± 1.8^a^	8.2 ± 3.2^a^	19.6 ± 8.2^a^	48.83 ± 2.3^a^	20.6 ± 1.7^a^	42.33 ± 2^a^	14.16 ± 1.1^a^	27.33 ± 20.6^c^	35.69 ± 33.17^c^	0.02 ± 0.1^a^	0.6 ± 0.2^b^	22.01 ± 22.5^a^
	300 mg/kg	5.73 ± 1.26^a^	12.2 ± 2.3^a^	25.4 ± 7.0^a^	43.8 ± 2.6^a^	21.03 ± 0.6^a^	49 ± 4.2^a^	19.72 ± 5.3^a^	53.02 ± 1.9^a^	44.7 ± 2.4^c^	0.33 ± 0.5^a^	0.4 ± 0.6^b^	21.61 ± 21.7^a^
	600 mg/kg	6.7 ± 1.4^a^	13.1 ± 1.2^a^	30.8 ± 5.0^a^	46.5 ± 2.2^a^	20 ± 3.03^a^	43.1 ± 4.5^a^	17.92 ± 6.1^a^	37.85 ± 33.3^b^	28.60 ± 27.81^b^	0.5 ± 0.1^a^	0.5 ± 0.5^b^	24.54 ± 23.6^a^

Neutral	Distilled H_2_O	6.76 ± 0.5^a^	14.5 ± 0.3^a^	31.70 ± 1.8^a^	47.1 ± 3.5^a^	21.5 ± 1.4^a^	45.7 ± 1.6^a^	11.38 ± 3.3^c^	25.71 ± 21.6^c^	51.44 ± 39.9^c^	0.61 ± 0.4^a^	3.6 ± 2.2^a^	18.64 ± 21.6^a^

The data are shown as mean ± SD (*n* = 3); values with the same superscript letter do not differ substantially at *p* ≤ 0.05. Red blood cells are represented by RBC, white blood cells by WBC, hematocrit by HCT, mean corpuscular content in hemoglobin by MCH, and mean corpuscular concentration in hemoglobin by MCCH. MO: leukocytes; BASO: basophils; EOS: eosinophils; NEU: neutrophils; LY: lymphocytes; Hb: hemoglobin.

**Table 4:** Effect of *B. micrantha* on biochemical parameters.

Group and doses	ASAT (serum)	ALAT (serum)	Protein (liver)
150 mg/kg	56.74 ± 14.23^c^	14.99 ± 3.63^a^	122.3 ± 16.83^a^
300 mg/kg	73.02 ± 9.90^b^	16.15 ± 3.63^a^	114.6 ± 4.69^a^
600 mg/kg	50.04 ± 9.83^c^	28.81 ± 0.29^a^	119.7 ± 11.09^a^
CQ 5 mg/kg	40.16 ± 1.26^c^	4.591 ± 2.34^b^	103.1 ± 25.74^a^
DMSO (1%)	73.78 ± 3.57^b^	53.98 ± 1.31^c^	132.7 ± 20.39^a^
Normal control	58.78 ± 7.27^a^	20.82 ± 5.32^a^	88.95 ± 3.48^b^

Alanine amino transferase is known as ALT and aspartate amino transferase as AST. The data is shown as mean ± SD (*n* = 3); values with the same superscript letter do not differ substantially at *p* ≤ 0.05.

**Table 5:** Effect of *B. micrantha* ethanol extract on MDA, NO, and glutathione levels.

Treatment	Glutathione (*μ*mol/g tissue)	MDA (*μ*mol/g tissue)	NO
600 mg/kg	1.707 ± 0.0601^c^	0.4519 ± 0.3152^a^	2.8983 ± 1.6464^a^
300 mg/kg	1.092 ± 0.1401^b^	0.6597 ± 0.04390^a^	2.955 ± 0.2606^a^
150 mg/kg	1.311 ± 0.1390	0.9598 ± 0.0876^a^	1.725 ± 1.39397^a^
CQ 5 mg/kg	1.055 ± 0.06365^b^	1.381 ± 0.1154	1.778 ± 0.5491^a^
DMSO (1%)	1.039 ± 0.07671^b^	2.214 ± 0.4424^b^	6.027 ± 1.258^b^
Normal control	1.543 ± 0.06652^c^	0.7874 ± 0.7341^a^	3.169 ± 0.2816^c^

The data are shown as mean ± SD (*n* = 3); values with the same superscript letter do not differ substantially at *p* ≤ 0.05.

## Data Availability

This research article includes every piece of data that was collected and examined.
